# Hepatorenal index for grading liver steatosis with concomitant fibrosis

**DOI:** 10.1371/journal.pone.0246837

**Published:** 2021-02-12

**Authors:** Fabio Lucio Stahlschmidt, Jean Rodrigo Tafarel, Carla Martinez Menini-Stahlschmidt, Cristina Pellegrino Baena

**Affiliations:** 1 School of Medicine Pontifical Catholic University of Paraná, Curitiba, Paraná PR, Brazil; 2 Marcelino Champagnat Hospital, Curitiba, Paraná PR, Brazil; University of Montreal, CANADA

## Abstract

**Introduction:**

Ultrasonography is widely used as the first tool to evaluate fatty liver disease, and the hepatorenal index is a semi-quantitative method that improves its performance. Fibrosis can co-exist with steatosis or even replace it during disease progression. This study aimed to evaluate the influence of fibrosis on the measurement of steatosis using the hepatorenal index.

**Materials and methods:**

This cross-sectional study included 89 patients with nonalcoholic fatty liver disease and in whom liver fibrosis was determined by ultrasound elastography. The Pearson’s correlation coefficient was used to compare between the results of the sonographic hepatorenal index and the quantification of steatosis using magnetic resonance spectroscopy as well the accuracy of detecting moderate to severe steatosis using sonography in two groups of patients: (A) without advanced fibrosis and (B) with advanced fibrosis. Advanced fibrosis was defined as a shear wave speed ≥ 1.78 m/s on ultrasound elastography. We calculated the area under the curve (AUC-ROC) to detect the ability of the hepatorenal index to differentiate light from moderate to severe steatosis in both groups. Moderate to severe steatosis was defined as a fat fraction > 15% on the magnetic resonance spectroscopy. The intra-observer variability was assessed using the Bland-Altman plot.

**Results:**

Among patients, the mean age was 54.6 years and 59.6% were women, 50.6% had a body mass index ≥ 30 kg/m^2^, 29.2% had moderate to severe steatosis, and 27.2% had advanced fibrosis. There was a correlation between steatosis grading by ultrasonography and magnetic resonance in group A (0.73; *P* < 0.001), but not in Group B (0.33; *P* = 0.058). The AUC-ROC for detecting a steatosis fraction ≥ 15% was 0.90 and 0.74 in group A and group B, respectively. The intra-observer variability for the hepatorenal index measurements was not significant (-0.036; *P* = 0.242).

**Conclusion:**

The hepatorenal index is not appropriate for estimating steatosis in livers with advanced fibrosis.

## Introduction

Fatty liver disease is a frequent condition worldwide [1] and the chronic liver damage in fatty liver disease can evolve from steatohepatitis to fibrosis [2] which in the advanced forms is defined histologically as stages F3 and F4 according to the Brunt scoring system [3,4]. The causes of mortality resulting from advanced liver fibrosis are mainly related to portal hypertension [5], and steatohepatitis is an independent risk factor for the development of hepatocellular carcinoma [6].

Ultrasonography is widely performed as a noninvasive test to screen steatosis because of its low cost and high availability, although, magnetic resonance spectroscopy has better accuracy [7–9].

The hepatorenal index is a semiquantitative measure used to estimate steatosis with ultrasonography [10–12]. Fibrosis can co-exist with steatosis and even substitute for it in the natural evolution of nonalcoholic fatty liver disease (NAFLD), probably interfering with the ultrasonographic evaluation of steatosis [13–16].

The detection of fibrosis in patients with NAFLD is crucial, as the presence of severe fibrosis signifying advanced chronic liver disease can predict complications and death [17]. The gross features of advanced fibrosis on ultrasound examination appear in a minority of patients with advanced chronic liver disease [18]. Fibrosis can be measured noninvasively using point shear wave ultrasound elastography with good histological correlation without the influence of steatosis [19–23].

The direct influence of advanced fibrosis in the measurement of the hepatorenal index requires further investigation.

### Objective

The objective of this study was to evaluate the impact of co-existing advanced fibrosis on the grading of steatosis using the hepatorenal index.

## Materials and methods

### Ethical considerations

This project was approved by the Institutional Review Board Ethics Committee (protocol number 2.211.249), and all patients signed the consent form.

### Study design

This was a cross-sectional study conducted from September 2017 to February 2019. Patients with suspected liver steatosis underwent abdominal ultrasonography with hepatorenal index measurement, liver ultrasound elastography, and magnetic resonance spectroscopy. The sample size was determined to be 10% of the patients referred to ultrasound examination in the hospital with steatosis or suspected to have steatosis in the last year before the start of the study.

The inclusion criteria were the presence of liver steatosis, age ≥ 18 years, and no chronic hepatitis B or C.

After an initial workup to exclude viral infection in the included patients, we interviewed the patients and reviewed their medical data to evaluate the exclusion criteria. The exclusion criteria were possible alcohol-related liver disease (alcohol ingestion > 120 g/week over the previous 3 months or history of alcoholism; hemochromatosis (patients with a liver iron concentration > 2 mg/g on T2 relaxometry resonance magnetic quantification); patients with biliary dilatation on ultrasound examination; patients with possible acute or subacute inflammation as a confounding factor for ultrasound elastography measurements defined as elevated transaminases levels > 5 times the normal reference levels in the previous 3 months; patients with possible hepatic congestion such as those with a history or signs of cardiac insufficiency or reduced compression of the inferior vena cava, pleural effusion, and predominant sonography B lines on lung ultrasound [24,25]; patients with a medical history of renal disease, a renal surgery, or the presence of right renal atrophy defined as < 8 mm in thickness; patients with heterogeneous hepatic liver infiltration visible both on ultrasound or on magnetic resonance segmentation; patients with technical limitations on which it is difficult to measure the hepatorenal index or perform ultrasound elastography, for example, the interposition of the colon to the liver or the kidney; patients with nonreliable ultrasound elastography defined as interquartile variation range > 0.15 m/s; claustrophobic patients; pregnant patients; and those with pacemakers, cochlear implants, aneurysm clips, implanted infusion pumps, or electro-stimulators [26].

We documented the presence of comorbidities as well as the patients’ anthropometric and sociodemographic information.

### Data collection

Each patient fasted for 4 h before the examination. First, the radiologist performed a comprehensive thoracic and abdominal ultrasound examination using the same ultrasound machine for all patients (S2000 HELX; Siemens, Erlangen, Germany). Next, a photo containing the longitudinal axis of the right kidney and the liver was obtained after automated gain adjustment in the dorsal decubitus position or in the left lateral decubitus position using the subcostal or intercostal window. A single focal zone was placed near the center of the image containing both the liver and the middle third of the kidney. For all examinations, the conditions were as follows: frequency of 4.00 MHz, dynamic range of 70, grayscale map “D”, the software Advanced SieClear^TM^ for spatial compounding at a level 5, and the software Dynamic TCE^TM^ for speckle reduction at a level “HIGH.”

**Fig 1:**
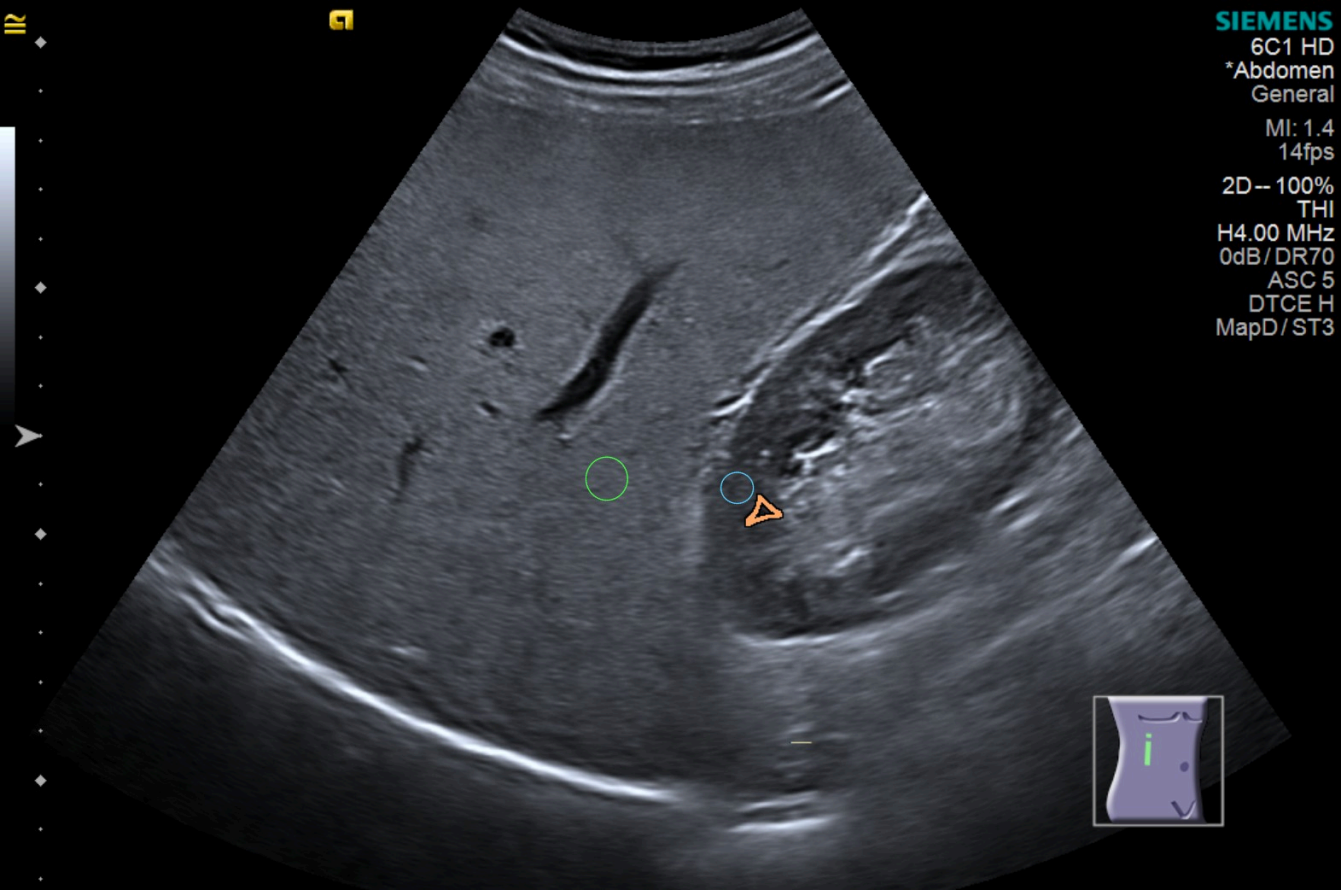
Hepatorenal index measurement in a fatty liver using the subcostal window. Green: Region of interest in the liver. Blue: Region of interest in the renal cortex. Orange: Renal pyramid. Author’s source.

**Fig 2:**
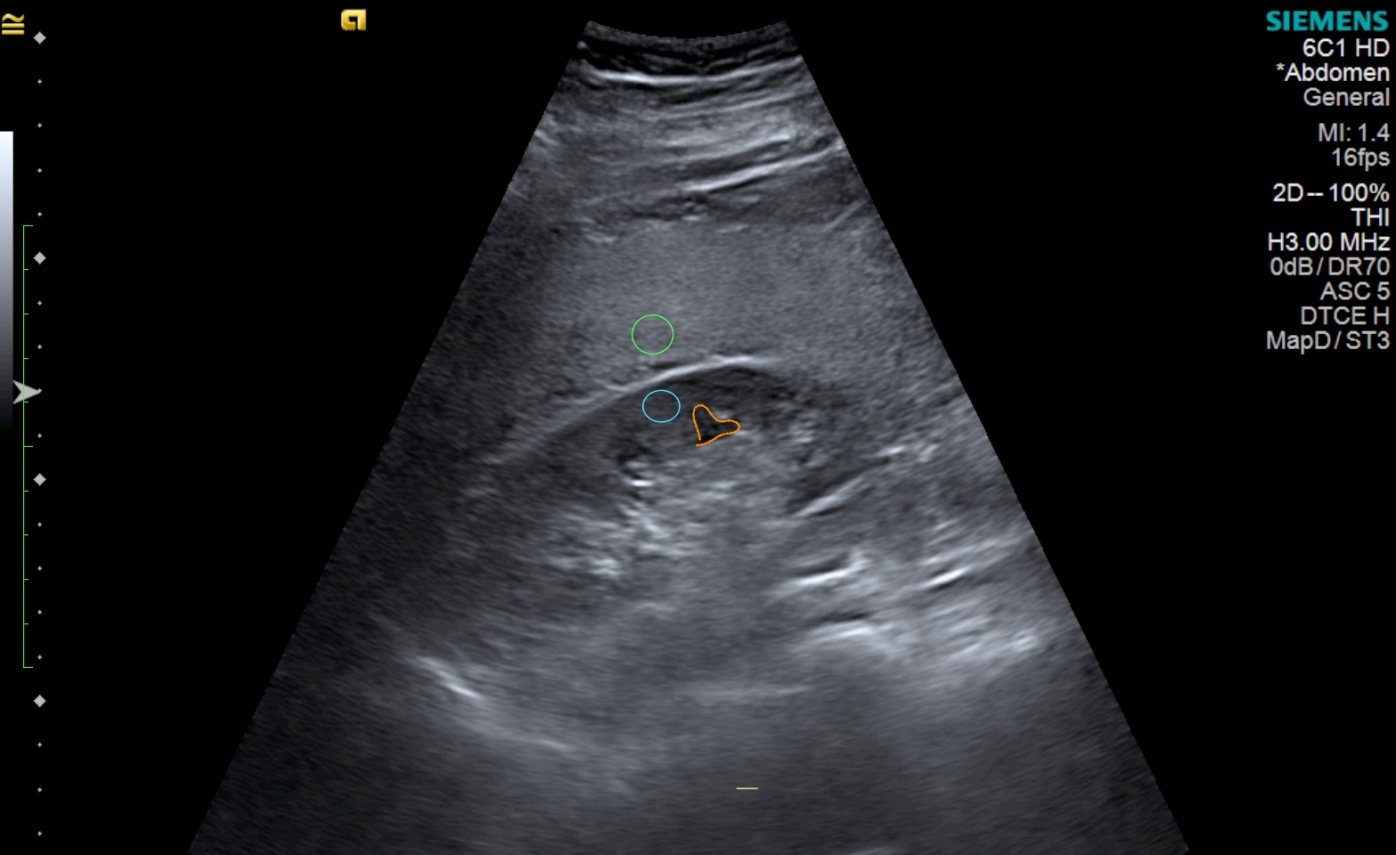
Hepatorenal index measurement in a fatty liver using the intercostal window. Green: Region of interest in the liver. Blue: Region of interest in the renal cortex. Orange: Renal pyramid. Author’s source.

Before examining the magnetic resonance results, one experienced abdominal radiologist calculated the hepatorenal index, which is the ratio between liver and kidney brightness on the same workstation for all analysis using Osirix MD version 1.6 (Pixmeo Sarl, Swiss) after taking a 0.10 to 0.30 cm^2^ region of interest (ROI) brightness measurement in the liver and renal cortex in the middle of the image. The site of the renal cortex ROI was outside or between the renal pyramids, not crossing a 2.0 cm limit above or below the focal zone line. The liver’s ROI also did not cross a limit of 2.0 cm from the focal zone line, also in the middle of the image. The measurements were not taken within or near the nodules and visible vessels (Figs 1 and 2). Three ROIs each were obtained for the liver and renal cortex [27].

The hepatorenal index was calculated for each sequence of ROI measurements. The difference between the closest indexes must be less than 0.20 hepatorenal index units, and the arithmetic mean of these closest values was then used for further analysis.

**Fig 3:**
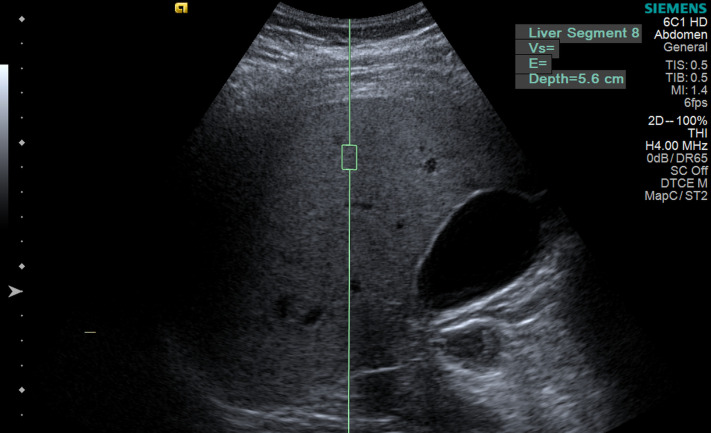
Ultrasound elastography measurement of liver stiffness. Author’s source.

Liver stiffness measurements were performed on the same day, after measurement of the hepatorenal index, using the same single ultrasound machine (S2000 HELX; Siemens, Erlangen, Germany) with an acoustic radiation force impulse point shear wave in liver segment VIII at 2–6 cm from the liver capsule with the patient in the dorsal decubitus position with the right arm abducted (Fig 3).

The group with advanced liver fibrosis was defined as having a shear wave velocity ≥ 1.78 m/s based on the study by Masato et al. [20], in which only patients with NAFLD with the same ultrasound model as that used in our study were included. The defined cutoff is more rigorous than those described in a local population [28] and in the recent update consensus from the Society of Radiologists in Ultrasound [29], which includes patients with chronic viral hepatitis. At least eight shear wave velocity measurements with an interquartile range of < 0.15 m/s were recorded [24].

**Fig 4:**
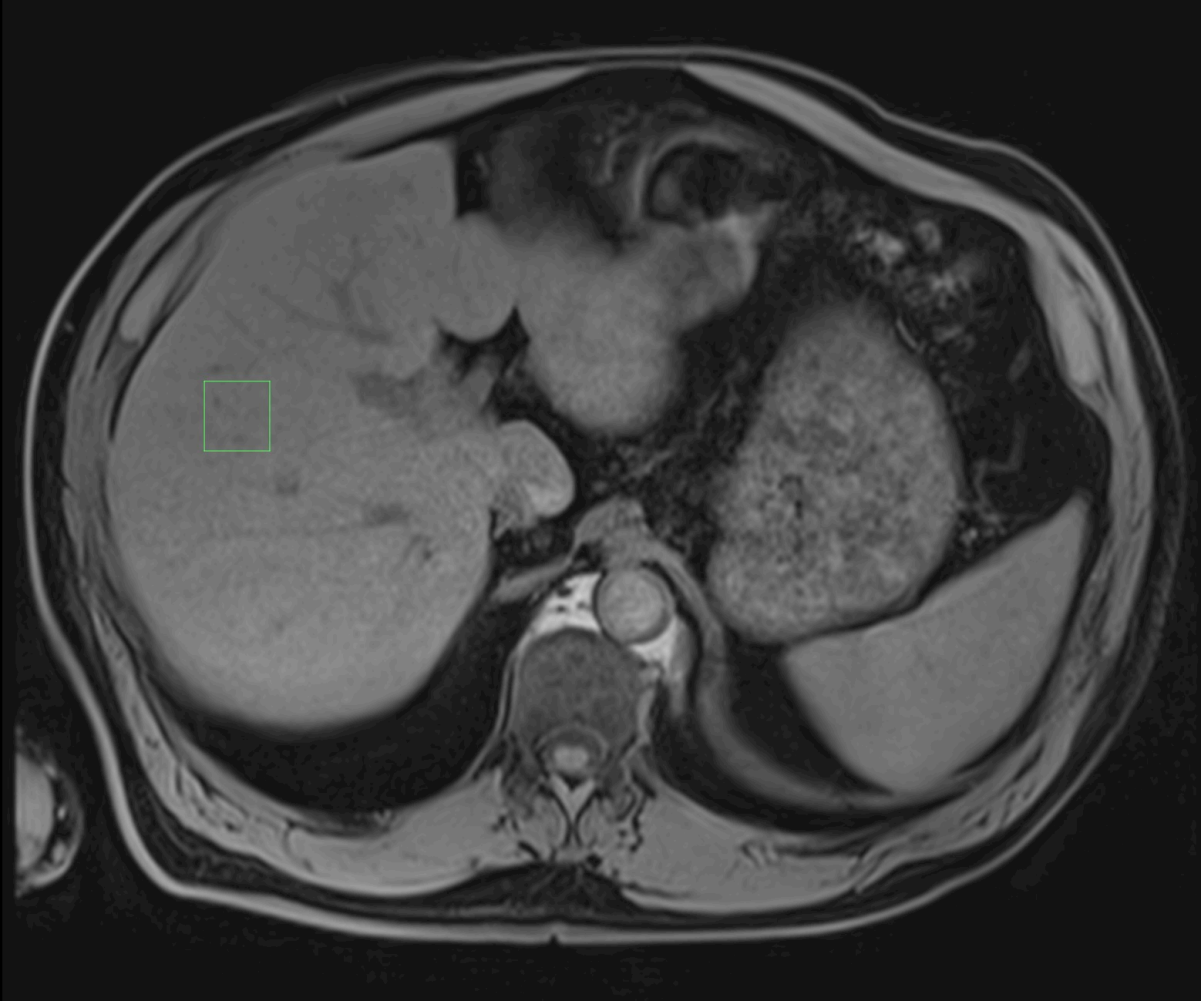
Spectroscopy voxel measurement in magnetic resonance. Author’s source.

**Fig 5:**
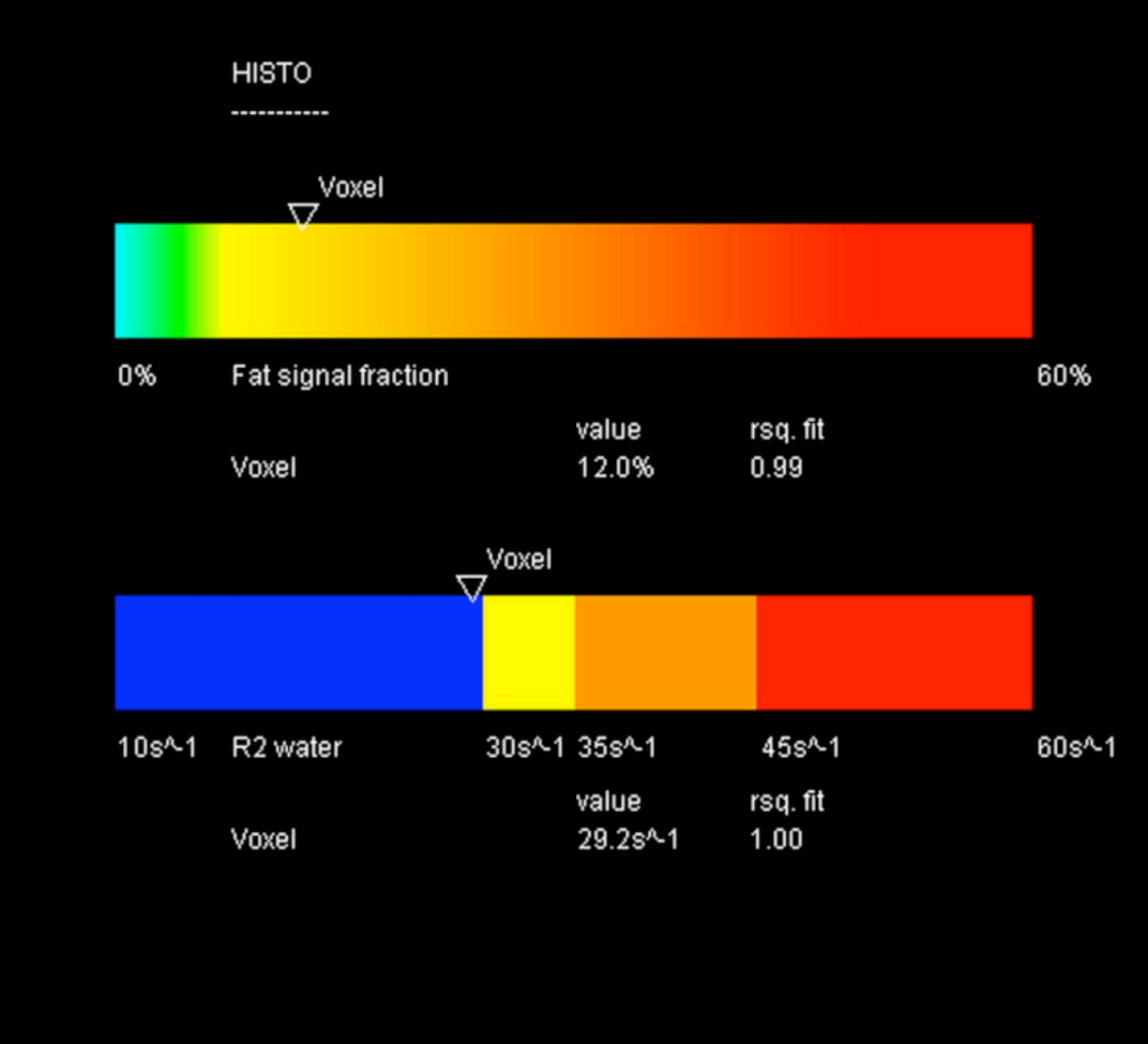
Fat fraction and iron relaxometry measured using LiverLab software. Author’s source.

After the ultrasound examination, the patient underwent a magnetic resonance examination in the same week using the same magnetic resonance imaging machine for all patients (1.5T MAGNETOM Aera; Siemens, Germany). The entire liver was first examined with a 16-channel body coil with a vibe e-Dixon sequence using a 400 mm field of view in the transverse direction, flip angle of 9°, and base resolution of 320 voxels. The iron concentration and fat fraction were measured using LiverLab software with the HISTO evaluation using spectroscopy single-voxel stimulated-echo acquisition mode of 3 × 3 × 3 cm^3^ in segment VIII, avoiding vessels and nodules. The acquisition used the following parameters: repetition time 3000 ms; echo time 12 ms, 24 ms, 36 ms, 48 ms, and 72 ms [30,31] (Figs 4 and 5).

Evaluation of the hepatorenal index was performed before analyzing the magnetic resonance imaging results to ensure that the radiologist was blinded at the time of the ultrasound examination. The ROI for the magnetic resonance spectroscopy was applied in the segment VIII by the technician and confirmed later by the radiologist.

### Statistical methods

We assumed a 95% detection rate of liver steatosis in patients without advanced fibrosis and 70% in patients with advanced fibrosis. We estimated a sample size of minimal of 72 considering 80% power and a two-sided alpha level of 0.05. To evaluate the association between the hepatorenal index (explanatory variable) and the fat fraction (response variable), we performed multilinear regression analysis and estimated the linear coefficients. We also performed a receiver operating characteristic analysis and calculated the area under the curve of the ability of the hepatorenal index to differentiate mild steatosis (fat fraction ≤ 15%) from moderate to severe steatosis (fat fraction > 15%) [32,33].

The groups were defined as follows: (A) without significant fibrosis (liver stiffness < 1.78 m/s) and (B) with significant fibrosis (liver stiffness ≥ 1.78 m/s).

We tested the correlation coefficient between the hepatorenal index and fat fraction. Linear regression models were built to analyze the correlation between the stiffness and the percentage of fat obtained through magnetic resonance imaging.

Sensitivity analyses were performed to verify the influence of comorbidities and the influence of inconsistent measures in the hepatorenal index defined as variations in mean value of the kidney ROIs > 10.0 brightness units’ value.

To access the hepatorenal index reproducibility, eighteen months from the first measurements, the same observer and an independent observer measured the hepatorenal index using the same images saved in the picture archiving and data system blinded to the previous results. Then, the intra-observer and inter-observer variability was calculated using the Bland-Altman test.

The data were analyzed using IBM SPSS Statistics v20.0 (Armonk, NY, IBM Corp.).

## Results

**Fig 6:**
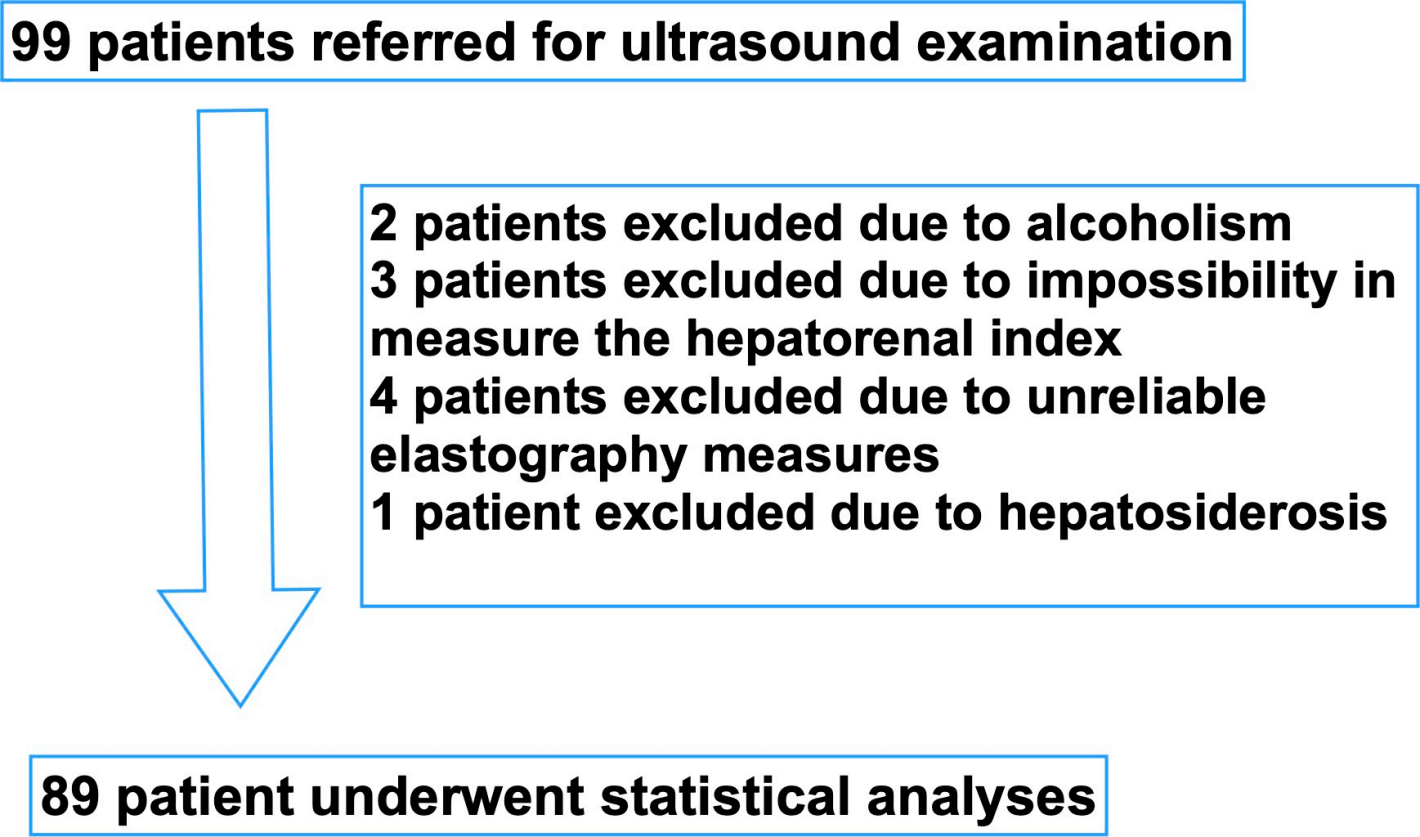
Flow chart of the studied population.

Ninety-nine consecutive patients were referred for the study, and 89 patients met the inclusion criteria. Two patients excluded due to alcoholism. Three patients excluded due to impossibility in the measure the hepatorenal index; for example, the interposition of colonic gas precluding the correct visualization of the kidney. Four patients excluded due to unreliable elastography measures, defined as shear wave velocity measurements with an interquartile range of > 0.15 m/s. One patient excluded due to hepatosiderosis (Fig 6). The mean age was 54.6 ± 12.4 years, mostly female and white, half of the patients were obese (body mass index ≥ 30 now kg/m^2^) and 38.2% had advanced fibrosis as shown in Table 1.

**Table 1:** Descriptive statistics.

Variable	Classification	General	Advanced Fibrosis		P*
			Without (n = 55)	With (n = 34)	
Age (years)		54.5 ± 12.5	53.5 ± 12.2	56.2 ± 13.0	0.334
	< 60	55 (64.7%)	37 (69.8%)	18 (56.3%)	
	≥ 60	30 (35.3%)	16 (30.2%)	14 (43.8%)	0.245
Gender	Male	34 (40%)	16 (30.2%)	18 (56.3%)	
	Female	51 (60%)	37 (69.8%)	14 (43.8%)	0.023
Ethnicity	White	71 (83.5%)	44 (83%)	27 (84.4%)	
	Latin	7 (8.2%)	4 (7.6%)	3 (9.4%)	
	Black	5 (5.9%)	3 (5.7%)	2 (6.3%)	
	Asian	2 (2.4%)	2 (3.8%)	0 (0)	-
Obesity (body mass index ≥ 30)	No	40 (47.1%)	31 (58.5%)	9 (28.1%)	
	Yes	45 (52.9%)	22(41.5%)	23 (71.9%)	0.008
Diabetes mellitus in medical records	No	62 (72.9%)	44 (81.1%)	20 (59.4%)	
	Yes	23 (27.1%)	10 (18.9%)	13 (40.6%)	0.043
Systemic arterial hypertension in medical records	No	45 (52.9%)	30 (56.6%)	15 (46.9%)	
	Yes	40 (47.1%)	23 (43.4%)	17 (53.1%)	0.502
Psoriasis	No	59 (69.4%)	37 (69.8%)	22 (68.8%)	
	Yes	26 (30.6%)	16 (30.2%)	10 (31.3%)	1
Fat percentage		12.5 ± 8.1	13.6 ± 9.0	10.7 ± 6.0	0.056
	≤ 15	59 (69.4%)	33 (62.3%)	26 (81.3%)	
	> 15	26 (30.6%)	20 (37.7%)	6 (18.8%)	0.090

*Student’s *t*-test for quantitative variables; Fisher’s exact test for categorical variables *P* < 0.05. The quantitative variables are described as mean ± standard deviations.

In group A, the hepatorenal index showed a correlation (R = 0.73; *P* < 0.001) with the fat fraction and accuracy in discriminating normal to mild versus moderate to severe steatosis with AUC = 0.90 (*P* < 0.001; 95% confidence interval (CI): 0.82–0.98).

In group B, the hepatorenal index showed a correlation with the fat fraction (R = 0.33; *P* = 0.058) and accuracy in discriminating normal to mild steatosis versus moderate to severe steatosis with an AUC = 0.74 (*P* = 0.074; 95% CI: 0.55–0.92).

**Fig 7:**
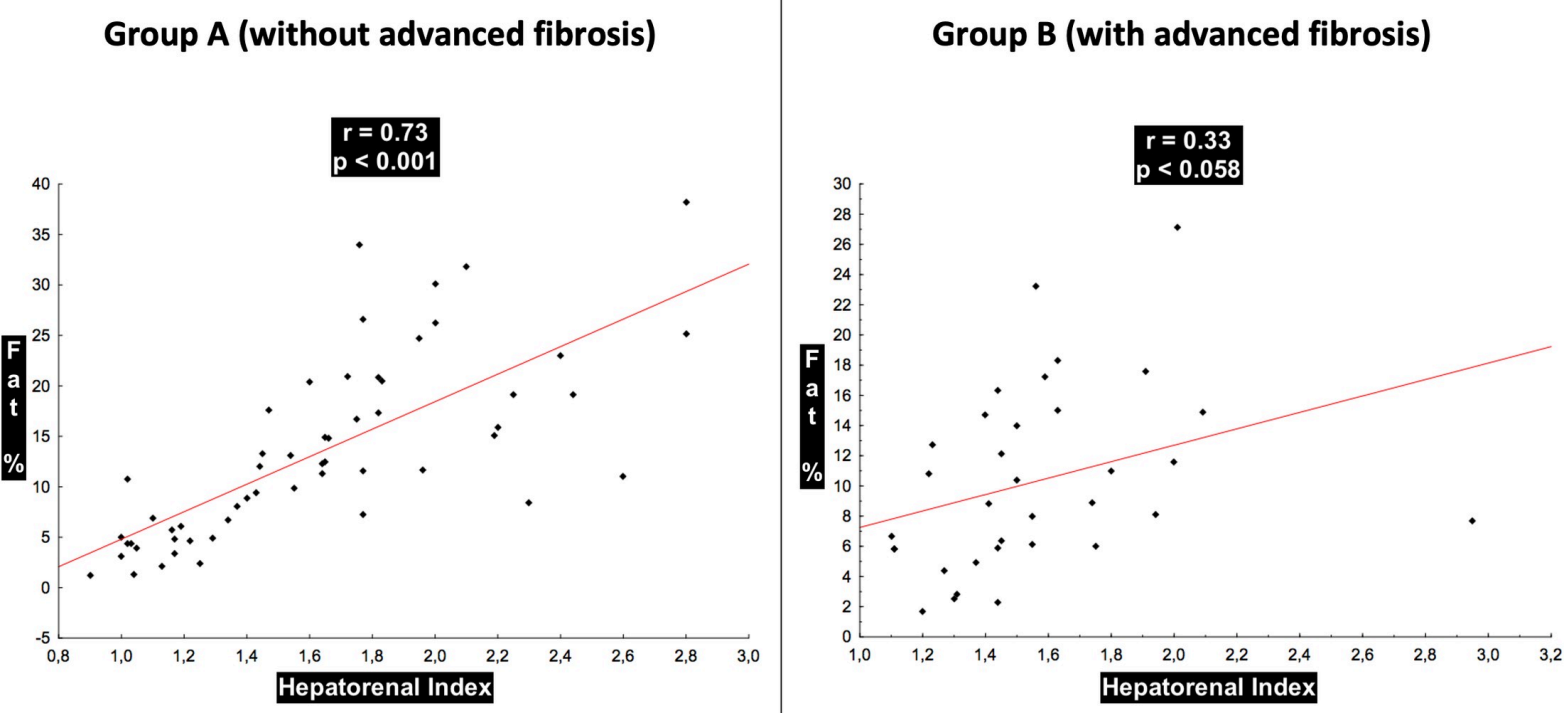
Distribution of the correlation between the hepatorenal index and the fat percentage of the groups considering the presence of advanced fibrosis.

The correlation between the hepatorenal index and liver fat fraction detected in magnetic resonance was highly positive in group A and low positive in group B using the rule of thumb for interpreting the size of a correlation coefficient [34] with statistical significance (*P* = 0.010) (Fig 7).

**Fig 8:**
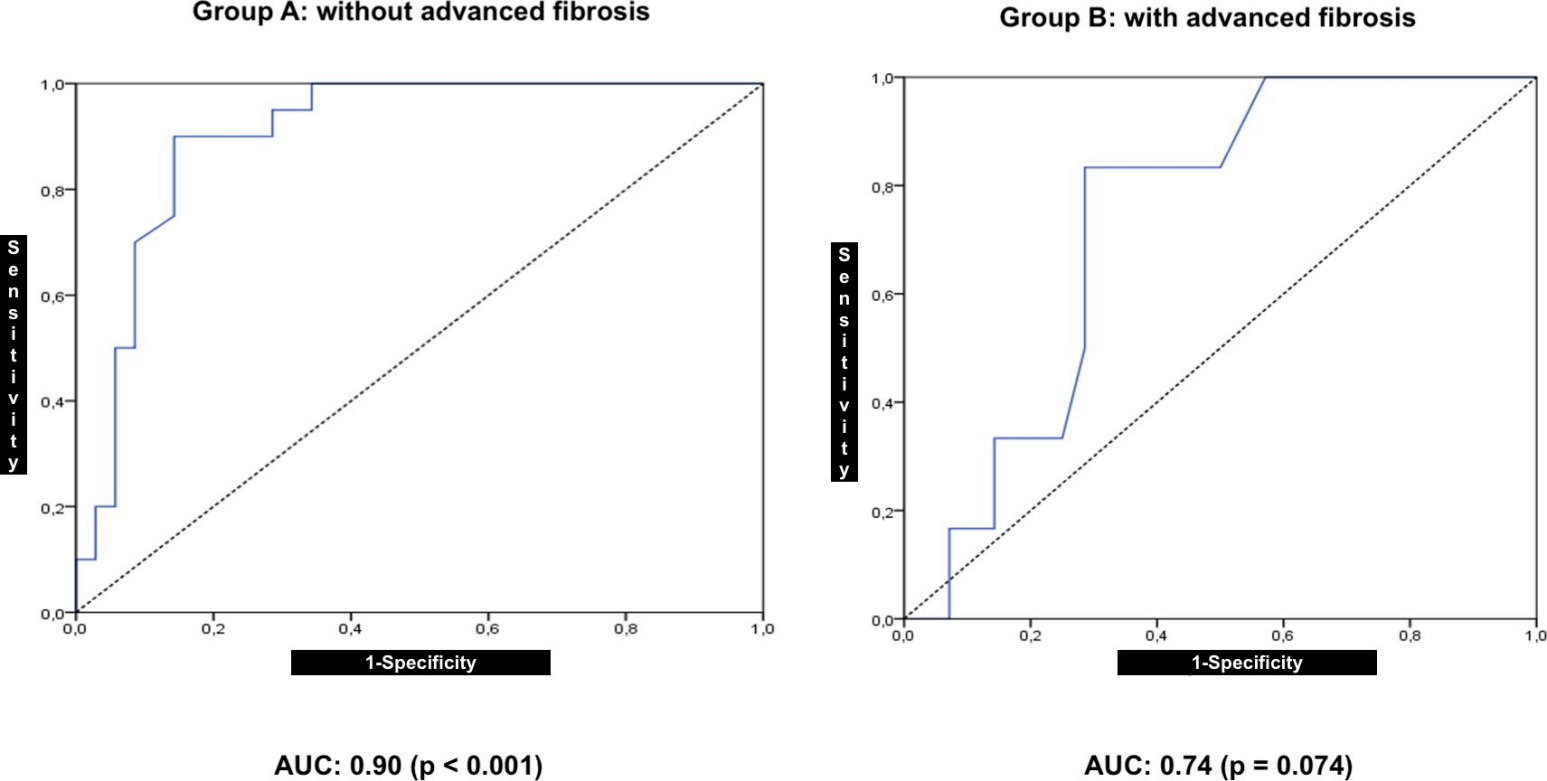
Receiver operating characteristic (ROC) curves for discrimination of moderate to severe fat percentage in the liver using the hepatorenal index in patient groups with and without advanced fibrosis.

The accuracy of the hepatorenal index to distinguish patients with a fat percentage ≤ 15% from those with a fat percentage of > 15% was excellent in group A but not comparable with that in group B, owing to the wide CI range (Fig 8).

**Fig 9:**
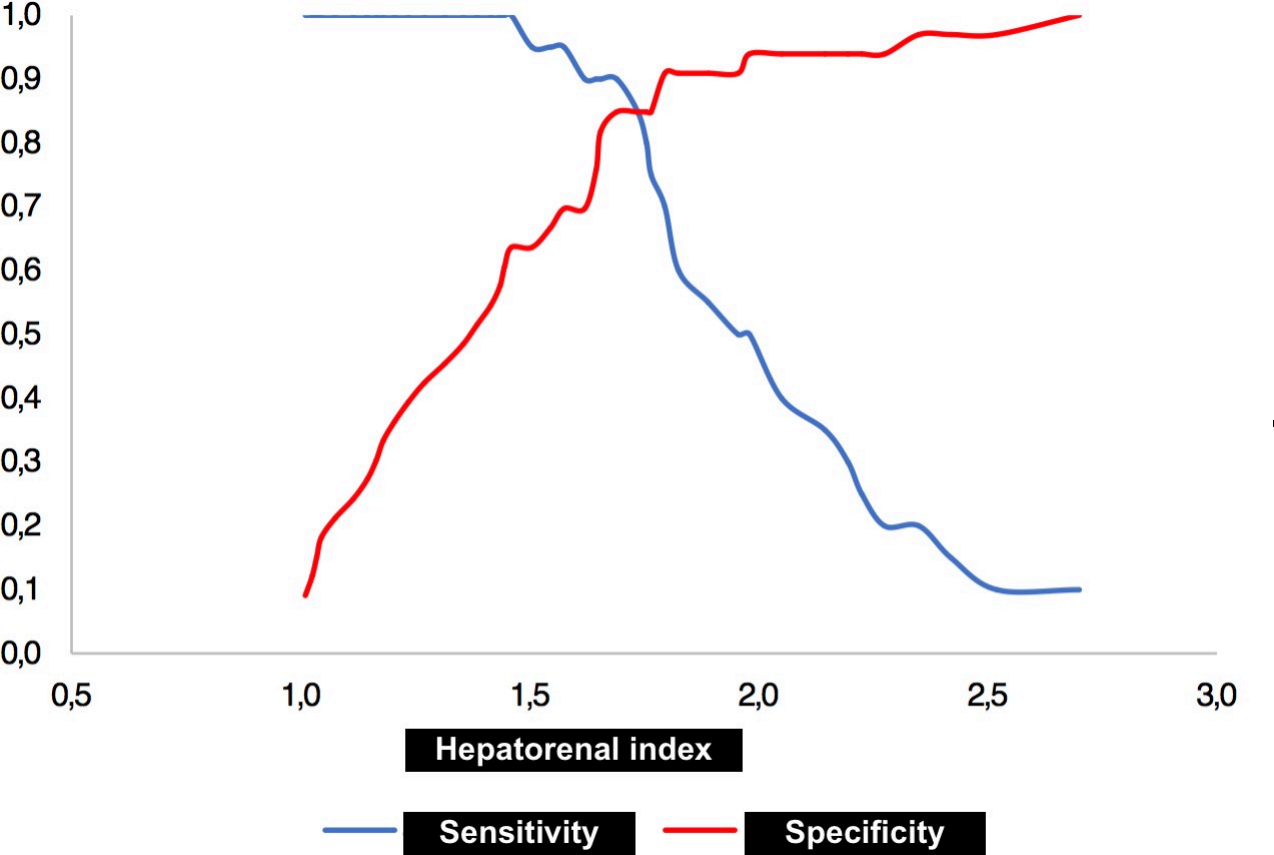
Two-graph receiver operating characteristic (ROC) curve with hepatorenal index cutoff value for discriminating moderate to severe fat percentage.

The indicated hepatorenal index cutoff value for discriminating a fat percentage > 15% was 1.69 with a sensitivity of 90% and a specificity of 85.7% (*P* < 0.001) (Fig 9).

A considerable proportion of patients with psoriasis were referred to our radiology department to investigate concomitant fatty liver disease. Thus, we performed a sensitivity test excluding these patients from the sample to verify whether the presence of psoriasis could be a confounding factor; however, the results did not change significantly (data not shown, *P* > 0.05 for all).

The renal cortex anisotropy can cause variability in the estimates of hepatorenal index depending on the site of the ROI measurements even if they do not cross a limit of 2.0 cm from the focal zone line. Four patients had variations in the mean value of the kidney ROIs > 10.0 units of brightness on the model. Then we performed a sensitivity test excluding these patients from the sample to verify whether the presence of the mentioned variability in ROI measurements could be a confounding factor; however, the results did not change significantly (data not shown, *P* > 0.05 for all).

The model to estimate the percentage of fat in the study population considering the hepatorenal index and shear wave velocity showed an adjusted R^2^ of 39.3% and intercept value of -5.13.

Formula: fat% = - 5.31–2.20 × (advanced fibrosis: 0 if no, 1 if yes) + 11.53 × (hepatorenal index).

**Fig 10:**
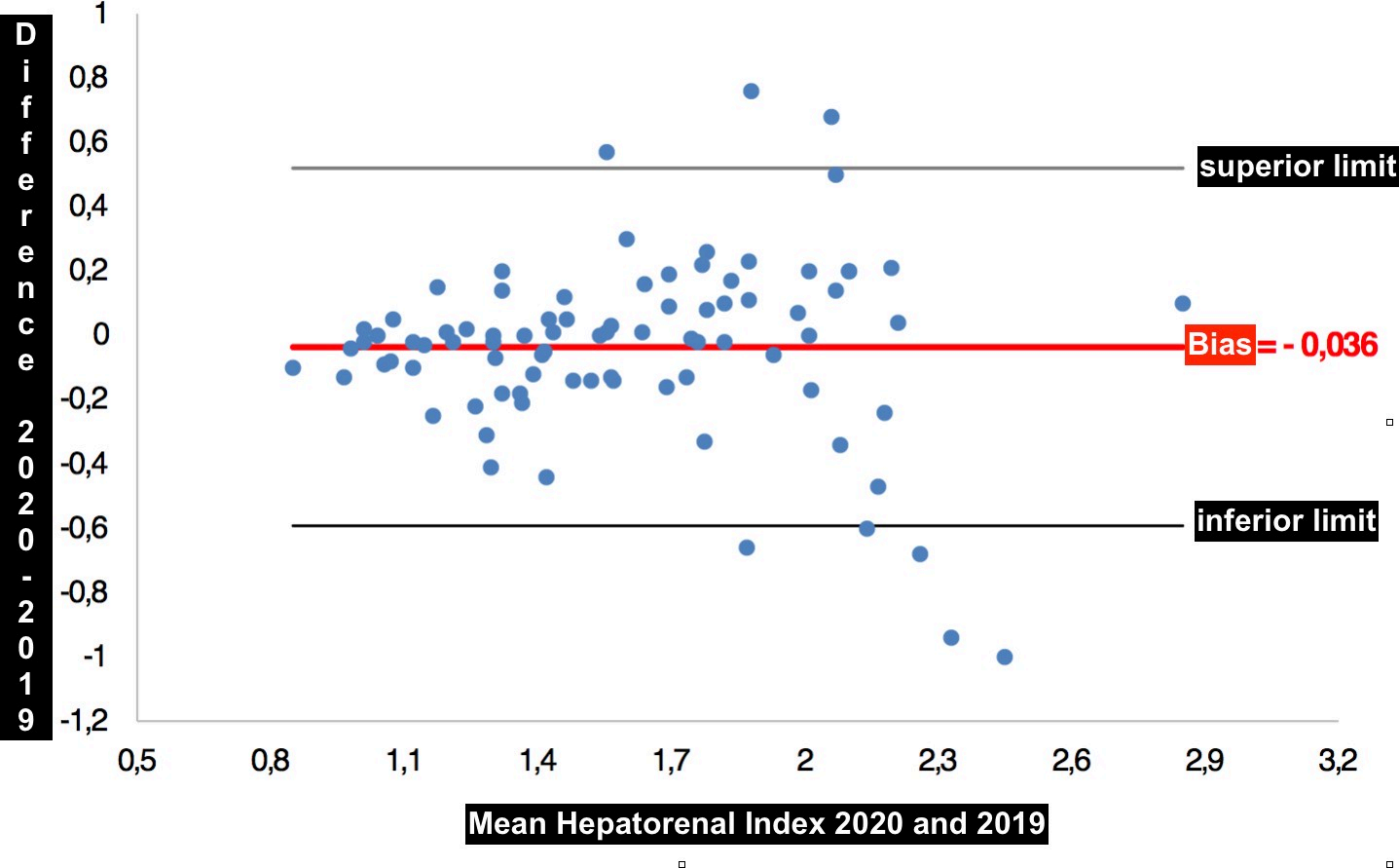
Bland-Altman graph showing the bias of -0.036 between the mean of the measurements made by the same observer.

**Fig 11:**
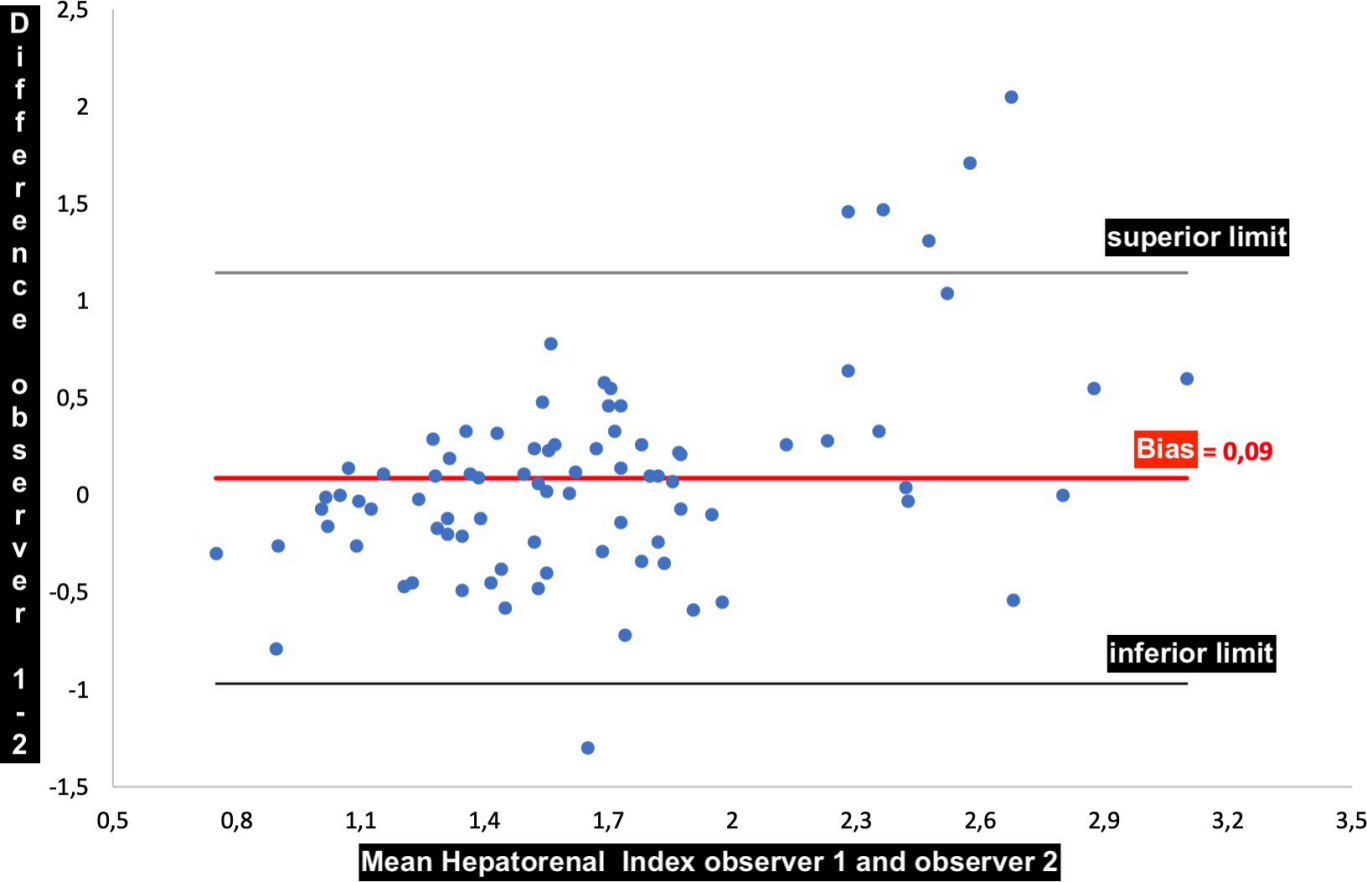
Bland-Altman graph showing the bias of 0.09 between the mean of the measurements made by the same observer.

There were no statistically significant intra-observer (*P* = 0.283) and inter-observer (*P* = 0.135) variations of hepatorenal index measurements using the Bland Altman analysis. The difference between the mean and median of the hepatorenal indexes was -0.036 and -0.010, respectively, with a standard deviation of 0.283 for the measurements made by the same observer (Fig 10). The difference between the mean and median of the hepatorenal indexes was 0.089 and 0.050, respectively, with a standard deviation of 0.539 for the measurements made by two observers (Fig 11).

## Discussion

Our findings indicate a significant influence of advanced fibrosis on the estimation of hepatic steatosis using the hepatorenal index. The present study compared steatosis grading by ultrasonography using the hepatorenal index in two groups of patients (with and without advanced fibrosis) with that using spectroscopy magnetic resonance. These results corroborate the hypothesis that fibrosis is a confounding factor in the ultrasound grading of steatosis [16] and provide substantial supporting evidence.

One explanation for our findings is that this observation could be related to the substitution of fat for fibrosis in the evolution of NAFLD [18], and in patients with advanced chronic liver disease, the hepatorenal index is not reliable for grading steatosis [14].

The accuracy of the hepatorenal index to discriminate mild from moderate to severe steatosis was high in patients without advanced fibrosis. Hence, our results are consistent with those of a previous study showing the hepatorenal index as a valuable tool for the detection and grading of fatty liver disease [8] in patients without advanced fibrosis.

Other studies that evaluated the influence of hepatic fibrosis on the subjective quantification of steatosis obtained conflicting results, such as the presence of interference [14] or a lack of interference [13,15]. Our study showed specific interference using the hepatorenal index. Possible explanations for the different results reported by Palmentieri et al. [15] and Petzold et al. [13] are that their studies evaluated a population with different etiologies of hepatic disease and a semiquantitative evaluation of steatosis through ultrasound examination of patients with fibrosis was not performed. Our study evaluated only patients with NAFLD and conducted a hepatorenal index ultrasonography evaluation of steatosis.

Ultrasound elastography in NAFLD has some limiting factors, such as the presence of severe acute inflammation, cholestasis, and concomitant liver diseases. Patients were carefully selected to exclude those factors from this study.

However, because we did not examine the patients histologically, low-grade inflammation could not be completely ruled out. Fat quantification by resonance included only one voxel in segment VIII; thus, the liver volume examination could have been sub-sampled. We performed a comprehensive liver ultrasound and obtained whole-liver resonance images using the Dixon technique. No studied patient had visual heterogeneity of fatty liver infiltration both on the ultrasound and magnetic resonance, which could indicate fat infiltration, severely affecting some liver segments and fat infiltration sparing of the others. Even so, not performing multi-segmental liver voxel spectroscopy is a limitation of this study.

Obesity, diabetes mellitus, and systemic arterial hypertension are common comorbidities in patients with NAFLD in the context of metabolic syndrome [17], and there is a high incidence of NAFLD in patients with psoriasis; however, whether this is an epiphenomenon, or an independent risk factor remains unknown [35].

The intra-observer and inter-observer agreements were high in our results, with means of measurements bias of -0.036 and 0.09, respectively. Data were gathered in a single private hospital, which limits the generalizability of our findings despite the main characteristics of our studied population being consistent with those of larger studies, such as the cohort profile of the ELSA-Brazil study [36].

The main strength of our study is that, to our knowledge, this is the first study to directly measure the interference of hepatic fibrosis in the measurement of the hepatorenal index. Considering that ultrasonography is widely used as the first tool to evaluate steatosis, the possibility of fibrosis associated with fat accumulation should be addressed before performing steatosis grading using the hepatorenal index to prevent misclassification.

Liver ultrasonography is a reliable and accurate test for the diagnosis of moderate to severe fatty liver, especially in patients without advanced fibrosis. For patients with fibrosis, semiquantitative estimation using the hepatorenal index is not reliable.

## Conclusion

The use of the hepatorenal index to estimate hepatic steatosis in the presence of concomitant fibrosis is biased. Therefore, advanced fibrosis should be excluded before taking into consideration the hepatorenal index.

## Supporting information

S1 Dataset (XLSX)
